# Dispatch accuracy of physician-staffed emergency medical services in trauma care in south-east Norway: a retrospective observational study

**DOI:** 10.1186/s13049-021-00982-3

**Published:** 2021-12-07

**Authors:** Martin Samdal, Kjetil Thorsen, Ola Græsli, Mårten Sandberg, Marius Rehn

**Affiliations:** 1 Department of Research, Norwegian Air Ambulance Foundation, Oslo, Norway; 2 Department of Anaesthesiology and Intensive Care/Air Ambulance Department, Drammen Hospital, Drammen, Norway; 3 Faculty of Medicine, University of Oslo, Oslo, Norway; 4 Pre-hospital Division, Emergency Medical Coordination Centre, Oslo University Hospital, Oslo, Norway; 5 Pre-hospital Division, Air Ambulance Department, Oslo University Hospital, Oslo, Norway; 6 Department of Health Studies, University of Stavanger, Stavanger, Norway

**Keywords:** Pre-hospital trauma care, Physician-staffed emergency medical services, Dispatch, Triage

## Abstract

**Background:**

Selection of incidents and accurate identification of patients that require assistance from physician-staffed emergency medical services (P-EMS) remain essential. We aimed to evaluate P-EMS availability, the underlying criteria for dispatch, and the corresponding dispatch accuracy of trauma care in south-east Norway in 2015, to identify areas for improvement.

**Methods:**

Pre-hospital data from emergency medical coordination centres and P-EMS medical databases were linked with data from the Norwegian Trauma Registry (NTR). Based on a set of conditions (injury severity, interventions performed, level of consciousness, incident category), trauma incidents were defined as complex, warranting P-EMS assistance, or non-complex. Incident complexity and P-EMS involvement were the main determinants when assessing the triage accuracy. Undertriage was adjusted for P-EMS availability and response and transport times.

**Results:**

Among 19,028 trauma incidents, P-EMS were involved in 2506 (13.2%). The range of overtriage was 74–80% and the range of undertriage was 20–32%. P-EMS readiness in the event of complex incidents ranged from 58 to 70%. The most frequent dispatch criterion was “Police/fire brigade request immediate response” recorded in 4321 (22.7%) of the incidents. Criteria from the groups “Accidents” and “Road traffic accidents” were recorded in 10,875 (57.2%) incidents, and criteria from the groups “Transport reservations” and “Unidentified problem” in 6025 (31,7%) incidents. Among 4916 patient pathways in the NTR, 681 (13.9%) could not be matched with pre-hospital data records.

**Conclusions:**

Both P-EMS availability and dispatch accuracy remain suboptimal in trauma care in south-east Norway. Dispatch criteria are too vague to facilitate accurate P-EMS dispatch, and pre-hospital data is inconsistent and insufficient to provide basic data for scientific research. Future dispatch criteria should focus on the care aspect of P-EMS. Better tools for both dispatch and incident handling for the emergency medical coordination centres are essential. In general, coordination, standardisation, and integration of existing data systems should enhance the quality of trauma care and increase patient safety.

**Supplementary Information:**

The online version contains supplementary material available at 10.1186/s13049-021-00982-3.

## Background

Physician-staffed emergency medical services (P-EMS) are integrated in the health system of most high-income countries [1]. The benefits of P-EMS are largely based on an assumed superiority of care, but the effects on morbidity and mortality remain debated [2–4]. In pre-hospital trauma care, specially trained physicians capable of performing advanced life support (ALS) procedures and the use of advanced point-of-care diagnostics may improve patient outcome [5–7]. Timely and accurate identification of patients predicted to benefit from P-EMS assistance, along with triaging patients to the designated hospital for definitive treatment, remain essential.

Selection of incidents that require P-EMS attendance, including the underlying dispatch criteria, is a designated area of research [8–10]. Advanced trauma care consists of a series of complex interventions involving heterogeneous populations and conditions, over various time intervals, by different providers with varying skill set, and in different organisational settings. Because of this complexity, trauma research is intricate and literature on the subject is characterised by numerous studies with large heterogeneity as well as varying quality of evidence [2, 11–14].

We aimed to evaluate P-EMS availability, the underlying criteria for dispatch, and the corresponding dispatch accuracy of trauma care in south-east Norway in 2015, to identify areas for improvement.

## Methods

### Setting

**Fig. 1:**
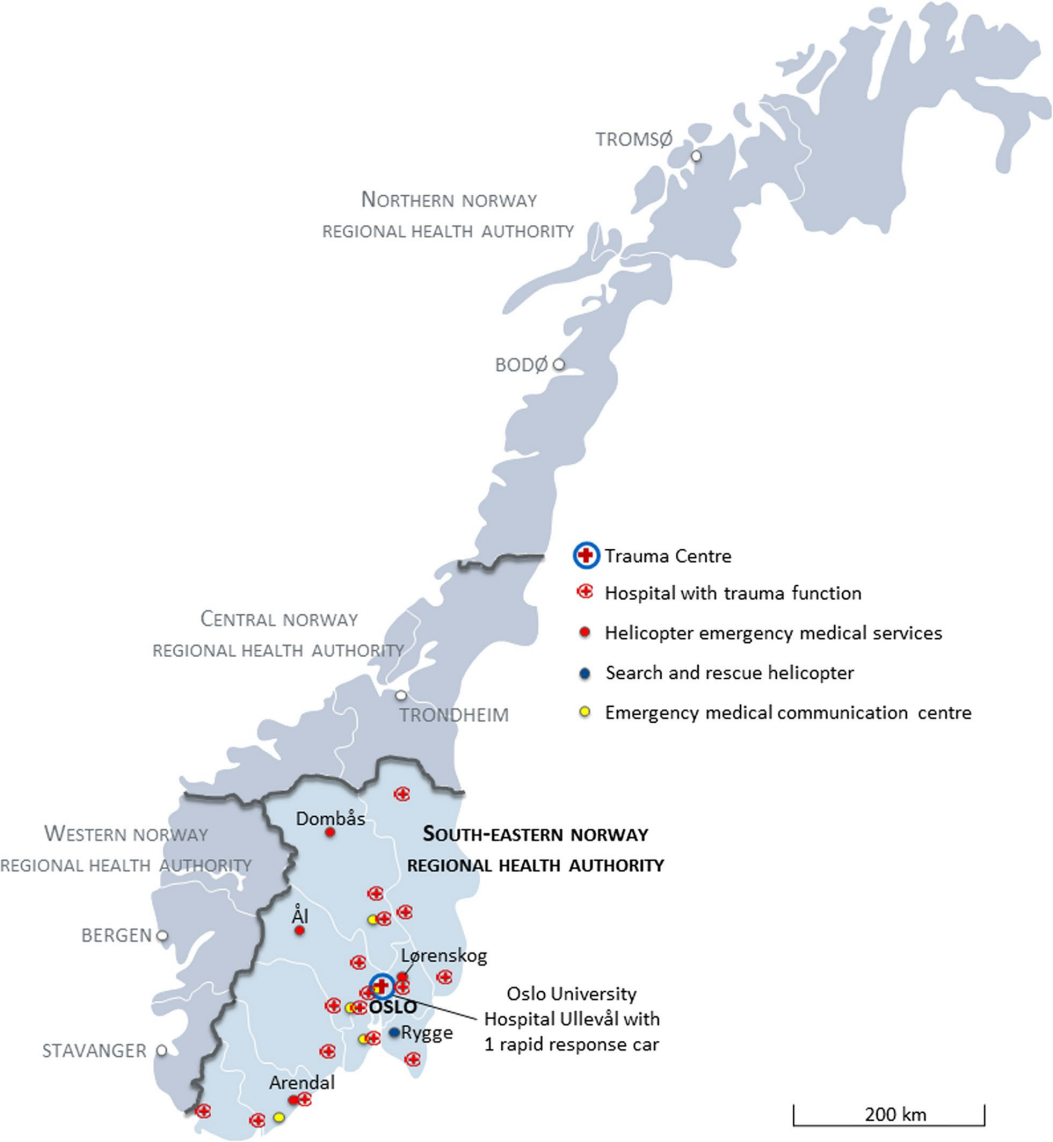
Map depicting selected Norwegian pre-hospital health resources in 2015

The study was conducted in south-east Norway with a catchment population of approximately 3 million (60% of Norway’s population) in 2015. The region housed 16 hospitals with trauma care functions (hereinafter referred to as trauma hospitals), in addition to the major trauma centre at Oslo University Hospital (OUH), Ullevål (Fig. 1).

### P-EMS in south-east Norway

Advanced pre-hospital treatment is provided by anaesthesiologist-staffed helicopter emergency medical services (HEMS), search and rescue (SAR) helicopters and rapid response cars (RRCs) without patient transport capacity. There are four HEMS bases in south-east Norway deploying five helicopters (two at Lørenskog), and one SAR base at Rygge (deploying one helicopter). HEMS respond to both trauma and medical emergencies and have the capacity of performing daylight rescue operations [15]. SAR perform offshore evacuations and rescue operations at all hours and can assist in both trauma and medical emergencies [16]. Both HEMS and SAR undertake primary and secondary missions (Table 1)*.*

**Table 1:** Mission categories

Primary mission	A mission where the patient is located out-of-hospital and transported to a designated level of care facility
Secondary mission	Inter-hospital transfer of a patient with the purpose of achieving a higher level of health care with more intensive/advanced treatment, due to acute injury or severe deterioration of condition
SAR	Search and rescue mission
Reversal	Transfer mission to a lower level of care facility, usually return trip to the local hospital/institution

All HEMS and SAR bases deploy RRCs in the vicinity of the base or when weather or technical issues prevent use of the helicopters. In addition, one stand-alone RRC is located at OUH Ullevål. All units are staffed by consultant anaesthesiologists, operating on a 24/7/365-basis. Since 2015, three more anaesthesiologist-staffed RRCs have been established in the region, however none operating at all hours.

### Emergency medical coordination centres (EMCC) and incident management

The region’s five EMCCs are staffed by specially educated nurses and EMS personnel using “Norwegian Index for Medical Emergencies” (Index), a criteria-based system for dispatch of EMS resources [17, 18]. The Index is organised in operative chapters, with corresponding sets of criteria and EMCC operator user guidance for accidents, medical conditions, and special circumstances (e.g. major incidents). Emergency calls are logged as incidents leading to dispatch of EMS resources (missions). All incidents are logged with a dispatch criterion chosen by the EMCC operator [19]. Norway does not have specific criteria for P-EMS dispatch [20].

**Fig. 2:**
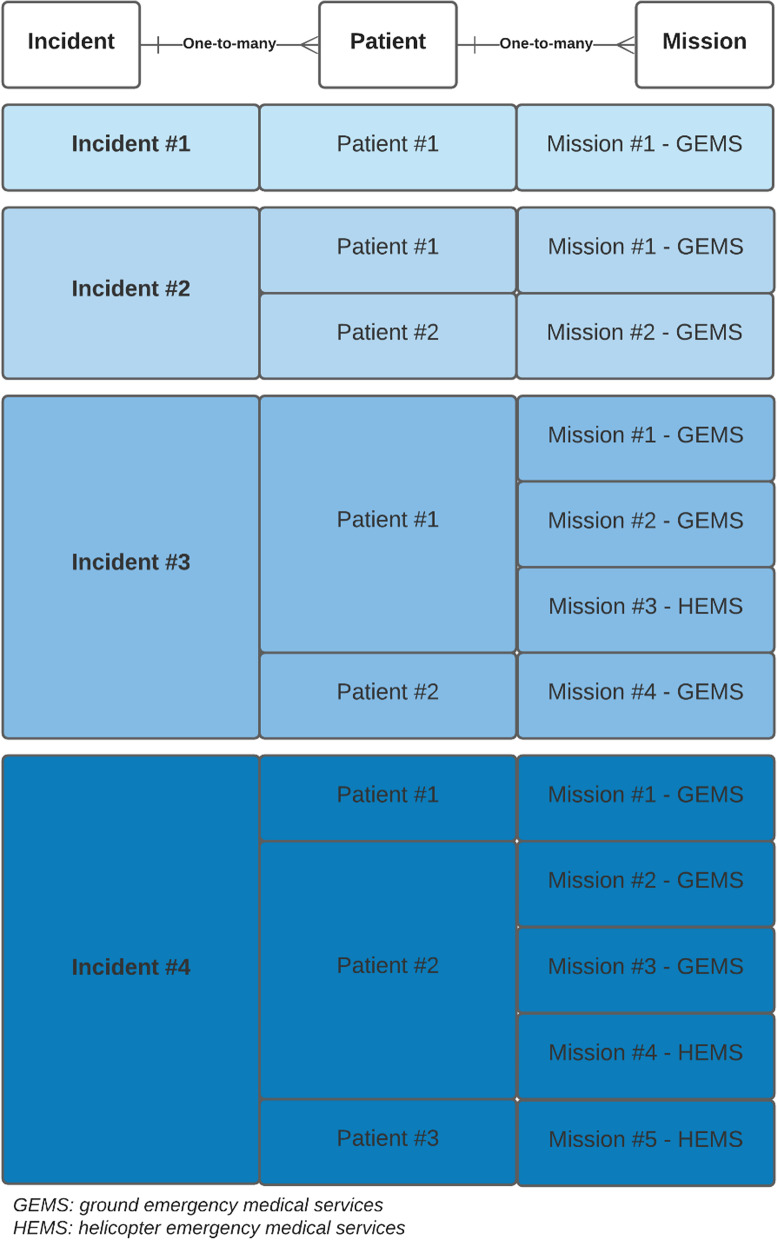
AMIS data structure, depicting the one-to-many relationship between incidents, patients, and missions. Traumas and accidents are recorded on an incident level with the associated number of patients and missions involved

AMIS (CSAM Health AS, Oslo, Norway) is the proprietary computer-aided dispatch system (CAD) applied by all the EMCCs in Norway. It contains incident log numbers, dispatch criteria and response level, operational descriptors, patient descriptors and general log data. Data are registered according to the sample structure depicted in Fig. 2.

### The Norwegian trauma system and the Norwegian Trauma Registry

A national trauma system was established in Norway in 2007 establishing a tiered-system with strategically located hospitals specialised in trauma care [21].

The Norwegian Trauma Registry (NTR) is a quality registry providing information on the extent and characteristics of severely injured patients, and the content and outcomes of the treatment provided [22].

### Study design

This is a retrospective observational study based on AMIS records from five different EMCC databases, medical records from one SAR and three HEMS databases, and records from NTR limited to patient pathways from south-east Norway.

### Data processing

The data from AMIS records was extensively cleaned due to poor quality and frequent incorrect input. Trauma incidents were initially identified by filtering on 94 selected dispatch criteria (Additional file 1). Primary missions were verified through manual assessment. Non-trauma incidents, trauma incidents containing only secondary missions and incidents without dispatch were excluded. The dispatched primary trauma incidents were then grouped into incidents with or without P-EMS involvement. HEMS rejections attributable to weather conditions, fatigue management regulations or technical issues were assessed as P-EMS involvement, indicating that EMCC considered P-EMS deployment in the dispatch phase. The incidents where further categorised as complex- or non-complex (Table 2).

**Table 2:** Conditions defining complex incidents

Condition		Data source
ALS procedures performed	Endotracheal intubation, pre-hospital or in the ED Tube thoracostomy, pre-hospital or in the ED Pre-hospital administration of TXA DCS (thoracotomy, laparotomy, extraperitoneal packing, re-vascularisation of extremity, interventional radiology, craniotomy, intracranial pressure monitoring)	NTR NTR P-EMS medical database, free-text field NTR
Initial GCS	Initial on-scene GCS ≤ 13 (we consider GCS of 13 as moderate TBI due to the higher incidence of ICI and poor outcomes in these patients compared to those with 14 and 15 [23])	NTR
Injury severity	NISS > 15	NTR
Major incident event	Index defines a major incident as “When the number of casualties exceeds the capacity of the EMS system” and contains 15 criteria in one dedicated chapter. All incidents logged with a major incident criterion were perceived as a complex, irrespective of actual or any injuries	AMIS, dispatch criteria

*ALS* advanced life support, *ED *emergency department, *TXA* tranexamic acid, *DCS* damage control surgery, *GCS* Glasgow coma scale, *TBI* traumatic brain injury, *ICI* intracranial injury, *NISS* new injury severity score

**Fig. 3:**
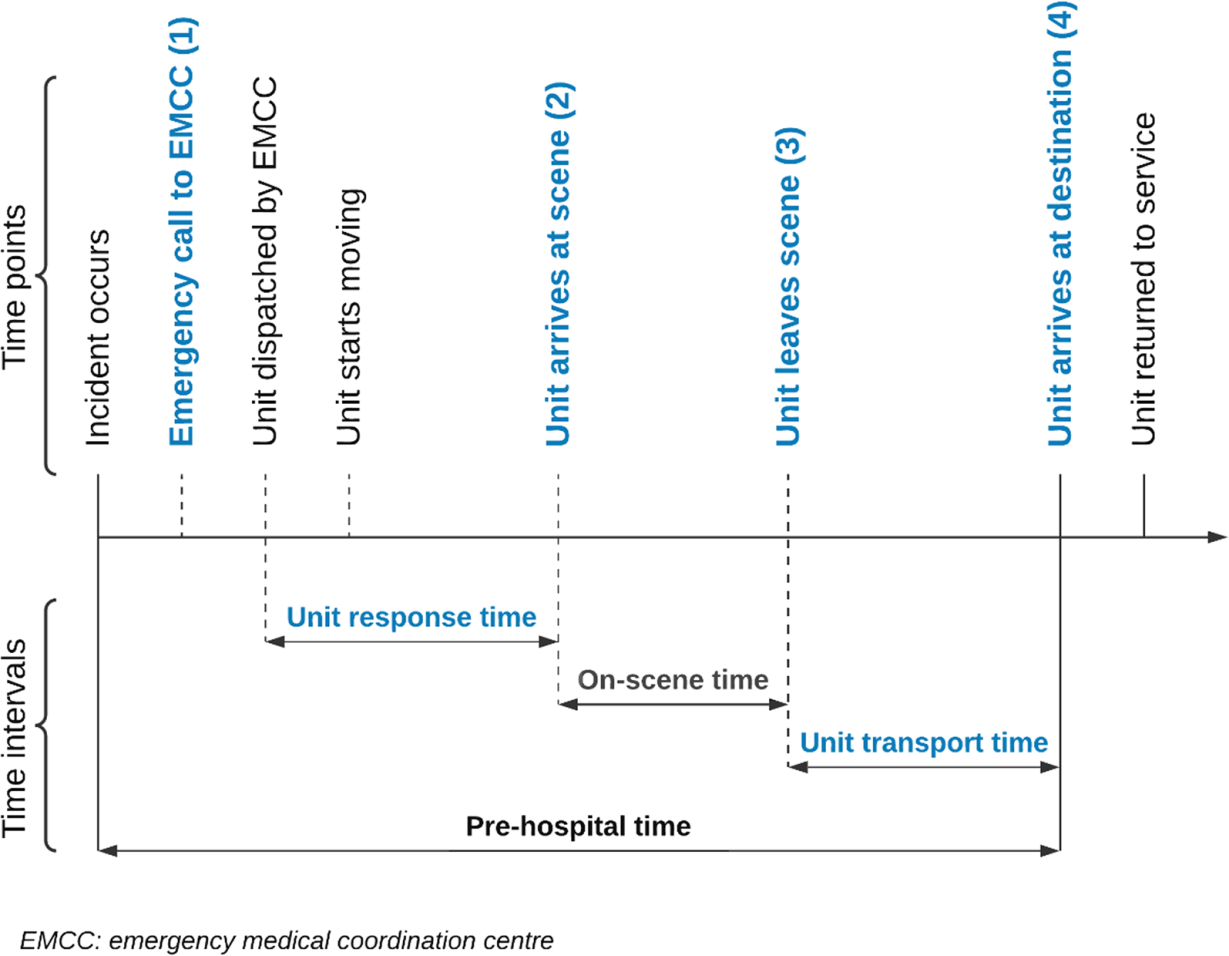
Pre-hospital timeline

Patient pathways from NTR were matched with AMIS incidents through the patient’s social security number (11-digit number), birth date, and the incident time. The initial match for patient pathways in NTR involved in complex incidents was just above 50% since numerous incidents were recorded with different dispatch criteria than the 94 criteria (Additional file 1) used to filter the first data extraction. In addition, both AMIS and NTR had missing or incomplete social security numbers. The accuracy increased to 86% after the unfiltered AMIS data was checked, and new matches manually verified. The remaining unmatched patient pathways in NTR were, unsuccessfully, attempted matched through various timestamp data (Fig. 3).

Incident complexity and P-EMS involvement were the main determinants when assessing the initial triage accuracy. Considering the regional differences in P-EMS coverage, undertriage was adjusted for the time aspect by examining P-EMS availability and response and transport times. The availability assessment was based on the registered time point of when P-EMS returned to service (Fig. 3).

If the unit was vacant or returned to service during the first 10 min after an incident was logged, it was considered available. Availability check for the RRC at OUH Ullevål, was restricted to incidents in the Oslo municipality (identified by municipality number).

Since Norway does not have specific criteria for P-EMS dispatch [20], a modified version of the Danish criteria of 2014 (Table 3), was applied to benchmark time expenditure [24].

**Table 3:** Modified Danish criteria for HEMS dispatch in 2014

HEMS should be considered	1. If the response time of the first pre-hospital unit exceeds 15 min and the nearest HEMS unit could reach the scene at least 10 min before this unit, or 2. In the event of a time-critical incident where the transport time to the nearest trauma hospital is expected to exceed 30 min, and HEMS deployment will reduce this time

The Danish criteria were chosen due to certain common features of our EMS systems and the ongoing Nordic efforts to identify and develop common quality indicators to develop comparable data [25]. We changed the term “nearest trauma centre” to “nearest trauma hospital” because of topographic differences. Denmark (43’ km^2^), with less than half of the surface area of south-east Norway (111’ km^2^), has four trauma centres. In comparison, south-east Norway has one trauma centre with response and transport times that can be considerably longer than in Denmark.

Due to the considerable portion of unmatched patient pathways in NTR, the results are reported in a confusion matrix (Table 4) with estimated ranges that account for the uncertainty of which incidents these pathways originate from. Unmatched pathways could initially originate from both complex and non-complex incidents, with or without P-EMS involvement.

**Table 4:** Confusion matrix of trauma incidents with dispatch of primary missions:

2015	Incident		Total (%)
	Complex	Non-complex	
*Dispatch*			
P-EMS	*A*	*B*	
	506–663 (2.7–3.5%)	1843–2000 (9.7–10.5%)	2506 (13.2%)
GEMS	*C*	*D*	
	641–798 (3.4–4.2%)	15,724–15,881 (82.6–83.5%)	16,522 (86.8%)
Total (%)	1147–1304 (6.0–6.9%)	17,724–17,881 (93.2–94.0%)	19,028 (100.0%)

*P-EMS* physician-staffed emergency medical services, *GEMS* ground emergency medical services, Undertriage: C/(A + C), Overtriage: B/(A + B)

Both the response and transport times and the distances were calculated by OpenRouteService (www.openrouteservice.org—Heidelberg Institute for Geoinformation Technology, Heidelberg, Germany) server by deploying data from OpenStreetMap (www.openstreetmap.org—OpenStreetMap Foundation, Cambridge, United Kingdom).

The Standards for Strengthening the Reporting of Observational Studies in Epidemiology (STROBE) guidelines were consulted [26].

## Results

**Fig. 4:**
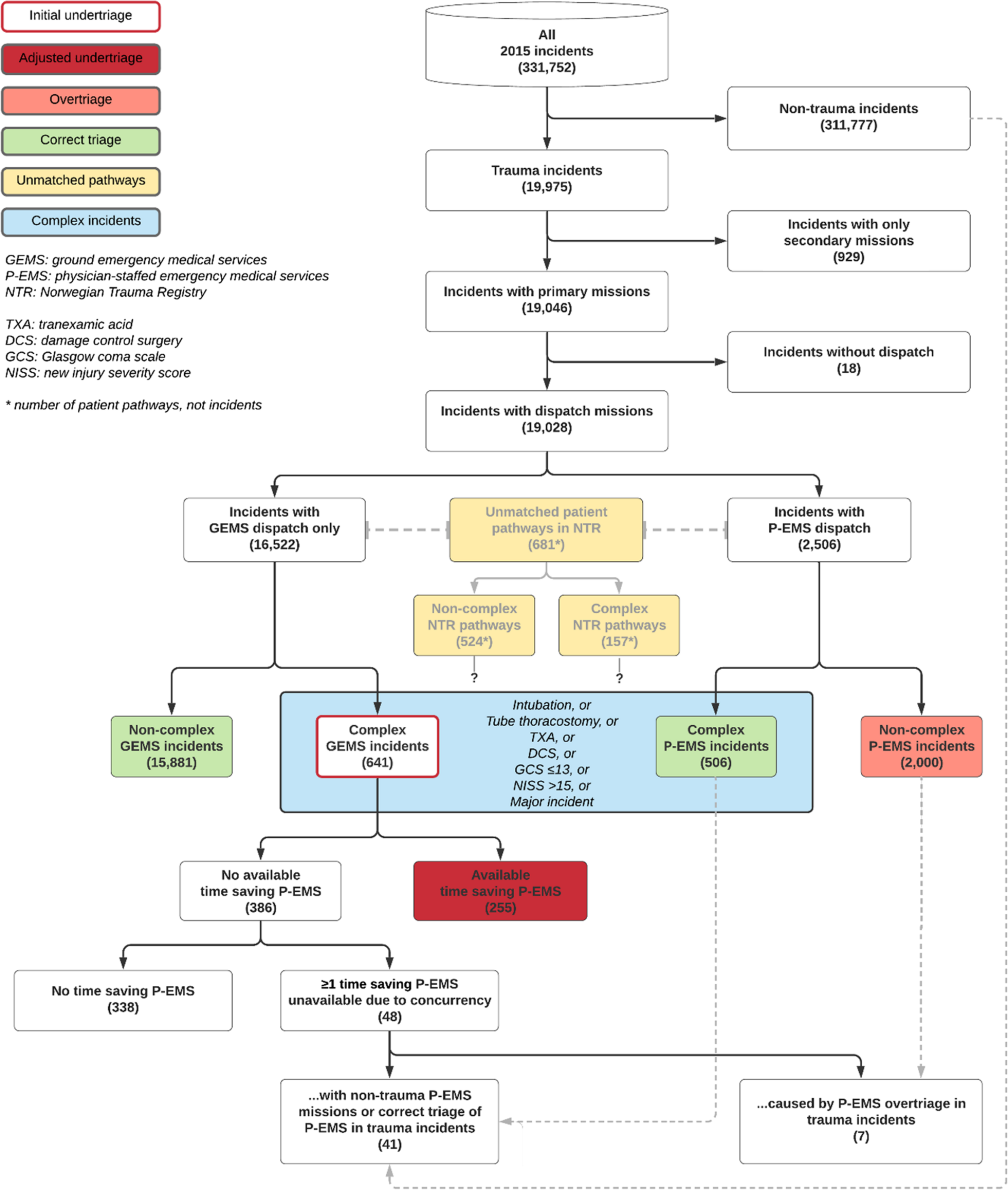
Track down of incidents, depicting categorisation, P-EMS involvement, triage and NTR pathways

In 2015, we identified 19,028 trauma incidents which lead to dispatch of at least one primary EMS mission in south-east Norway (Fig. 4). Among these, P-EMS were involved in 2506 (13.2%) (Table 4).

The calculated initial range of undertriage was 49–61%. Among the incidents categorised as undertriage, no P-EMS were time saving in 338 incidents (42–53%). When adjusted for availability and response and transport times, the range of undertriage decreased to 20–32%. The calculated range of overtriage was 74–80%. We identified seven trauma incidents where overtriage led to missed tasking, depriving P-EMS assistance to other complex incidents. The most vulnerable P-EMS unit to trauma overtriage was the RRC at OUH Ullevål which was affected in five of the cases.

The general P-EMS readiness in the event of complex incident ranged from 58 to 70%.

Among the 4,916 patient pathways from south-east Norway in NTR, we were unable to match 681 (13.9%).

**Fig. 5:**
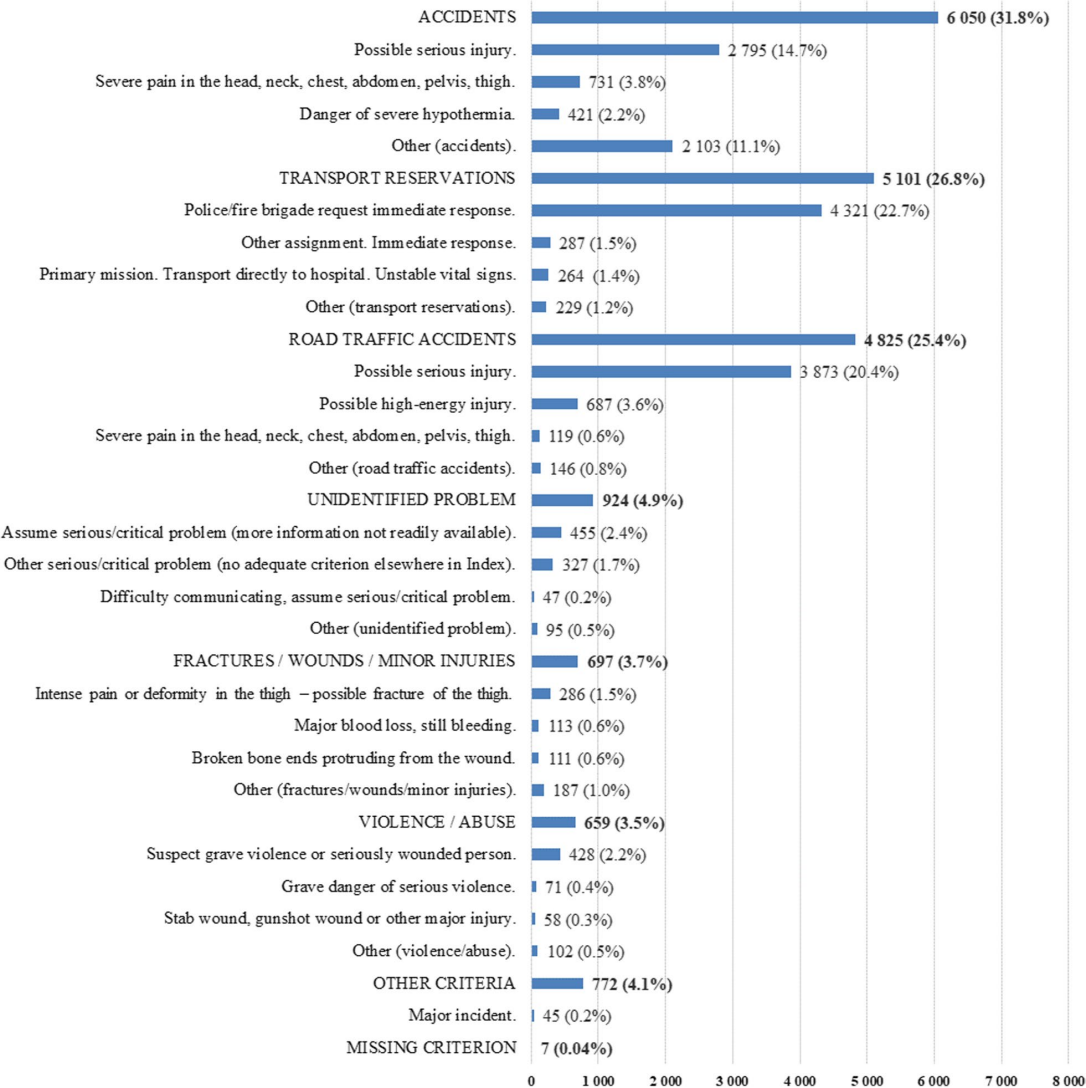
Dispatch criteria overview

The most frequent dispatch criterion was “Police/fire brigade request immediate response” from the group “Transport reservations” recorded in 4321 (22.7%) of the incidents (Fig. 5). Criteria from the groups “Accidents” and “Road traffic accidents” were recorded in 10,875 (57.2%) incidents, of where “Possible serious injury” accounted for 6668 (61.3%) of them. Criteria from the groups “Transport reservations” and “Unidentified problem” were recorded in 6025 (31.7%) incidents.

A criterion from the group “Major incident” was recorded in 45 (0.2%) of the incidents, of where “Fire/explosion—on land” accounted for 21 of them.

In 513 (2.7%) trauma incidents, two or more EMCCs were involved. Among these incidents, differing dispatch criteria were recorded in 346 (67.4%) of the cases, whilst different incident and/or mission categories were recorded in 121 (23.6%) and 61 (12.0%), respectively.

## Discussion

We found that the undertriage of P-EMS dispatch in south-east Norway ranged between 20 and 32% when adjusted for availability and response and/or transport times. Our key findings indicate that triage decisions and dispatch practise of P-EMS have several potentials for improvement. Dispatch criteria are vague, and source data are inconsistent.

### Triage

No consensus on accepted levels of triage precision exists [27–29]. The American College of Surgeons Committee on Trauma (ACS-COT) reflects that an adequate percentage of overtriage is in the range of 25–35% [30]. Overtriage decreases P-EMS availability by depriving other victims’ access to advanced pre-hospital critical care. Furthermore, it carries unnecessary risk, financial costs, and personnel fatigue. Reducing the substantial level of overtriage (74–80%) of P-EMS in south-east Norway remains an obvious area for improvement.

P-EMS undertriage is of more concern because it may result in preventable mortality or morbidity [31]. ACS-COT has defined < 5% as the acceptable level of undertriage [30]. Wisborg et al. [32] found that only half of the severely injured trauma victims in Norway were reached by P-EMS in 2013. Our initial calculated range of undertriage (49–61%) is in line with this finding. When adjusted for availability and response and transport times, the undertriage range decreased to 20–32%. In areas with scarce P-EMS coverage or when the nearest P-EMS unit was unavailable, vacant units proved non-eligible due to long response or transport times distance in 30–42% of the times. This suggests that the general P-EMS readiness in the event of complex incidents is too low. Several local RRCs have emerged since the study data was collected. The effect on both P-EMS availability and triage remains to be seen and should be addressed in future studies. RRCs are less costly as well as easier to establish and operate. Assuming an adequate volume of missions, RRCs seem like a promising adjunct to HEMS in areas with scarce HEMS coverage, provided concurrent dispatch of RRC and HEMS for primary response to scene and secondary transfer to trauma centre, respectively.

### Dispatch

Optimal P-EMS dispatch remains controversial. No globally recognised guidelines exist, and the evidence base remains weak. In a wealthy nation like Norway, with a well-equipped prehospital system, it is remarkable that specific criteria for P-EMS dispatch are missing. It is outside the scope of this study to discuss possible explanations to this shortcoming. However, P-EMS is a limited and costly resource, and optimal utilisation with focus on accurate dispatch guidelines should continuously be on the agenda for decision-makers of any EMS-system. In Denmark, P-EMS dispatch is based on selected criteria [both anatomical, physiological, and mechanisms of injury (MOI)] and incident type, in combination with time criteria. The Danish set of criteria is a translation of the Norwegian Index, adapted to Danish conditions. Whether anatomical or physiological criteria or MOI form the best basis of dispatch, remain debated [11, 33, 34]. Further, time criteria only concern the transport aspect of P-EMS. We suggest focusing on the care aspect of P-EMS, by including the competency and quality dimension provided by the physician in future guidelines, to improve dispatch accuracy.

Our set of criteria defining complex incidents warranting P-EMS has deliberately focused on documented items like ALS procedures, medication, and NISS. Initial GCS was included as the only physiological parameter given that a relatively uniform practise in reporting has been established, and because it extensively used to classify traumatic brain injury (TBI) into levels of severity and prognosis [35, 36]. *Major incident* was included since the public anticipates “exhaustive use” of resources in such events. The incidence of 45 major incidents, based on dispatch criteria solely, in 2015 is however not compatible with the findings of Johnsen et al. [37], who identified 50 major incidents in Norway from 2000 to 2016. This sustains the need of commonly developed definitions as well as training of personnel to achieve an agreed understanding of the term.

We found that one third of the dispatch criteria related to the pending nature of the incident, like “Transport reservation” and “Unidentified problem”. Another third was logged with the criterion “Possible serious injury” from the groups “Road traffic accidents” and “Accidents”. In total, more than two thirds of the dispatch criteria are non-specific or vague. In literature, dispatch and triage criteria have traditionally been categorised according to their nature as physiological, anatomical or MOI [11], but none of these categories are attributable in this context. This complicates the inclusion of dispatch criteria in e.g. regression analysis to detect variables that correlate with severe traumas. In general, we believe removal of criteria like “Possible serious injury” or vague criteria from chapters like “Transport reservation” or “Unidentified problem” is fundamental in future efforts to make the criteria more accurate.

### Data quality

Through the data analysis we learned that the inconsistencies in the pre-hospital data were considerable. The EMCCs utilise proprietary CAD clients, without a common registration platform or standardised registrations options. This results in separate names for units, institutions, and categorisation in general. Further, dispatch of P-EMS is subject to unitary coordination causing overlap in catchment/operating areas and EMCC interaction. The latter allows for double recording by the EMCCs involved, carrying divergence in time registration, scene coordinates, dispatch criteria and mission-/incident categorisation. All these factors strongly confuse data compilation, rendering data analysis difficult and complicate scientific research efforts in the field. In addition, the Index is applied to a variable extent, both on an individual operator level and amongst the EMCCs [38, 39].

In 2015, 1.2% of the trauma patients in Norway were brought to a trauma hospital in private vehicles without involvement of EMS and incident recording in AMIS [32]. Still, the main reasons for the non-match between patient pathways in NTR and AMIS incidents, are differences in the recording of patient ID and time logistics among the systems. A relatively easy solution to this problem would be to integrate the CAD incident log number as one data field in NTR.

### Limitations

Data originates from 2015, due to several reasons. A lengthy legal process concerning the establishment and launch of the NTR, and subsequent technical issues in terms of data collection and distribution caused a significant delay in study start, analysis and completion. We were not able to obtain medical data from the HEMS base at Dombås. Consequently, we have no data on TXA administration from that base. However, the actual incidents may well have been re-captured if they were categorised as complex through relating NTR records.

The grading of children’s level of consciousness may be inaccurate, since GCS is the only parameter registered in NTR.

Since 2015, there has been a minor syntactical revision to the Index. This revision has neither altered the structure of the Index nor dispatch practise in general, and we do not consider it to influence on our findings. Given the retrospective design, the study is limited to data variables in existing registries. The structural inconsistency and poor data quality of AMIS is substantial, rendering more in-depth data analysis challenging. The value range of the “true negatives” (non-complex incidents without P-EMS involvement; D-cell of Table 4) is in that respect “constructed”, since the extraction of trauma incidents originate from data based on the selected criteria-filter (Additional file 1).

Also, NTR records from the implementation phase in 2015 is inconsistent. Although these factors complicate synthesis of data between the registries, it remains difficult to estimate the actual impact on our findings.

In general, the external validity of the study is limited by the characteristics of the Norwegian trauma system and similar systems abroad.

## Conclusions

P-EMS dispatch in trauma care in south-east Norway suffered from an overtriage between 74 and 80% and an undertriage between 20 and 32%, when adjusted for concurrency and response and transport times. The general P-EMS readiness in the event of complex incident ranged from 58 to 70%. Index criteria are too vague to facilitate accurate P-EMS dispatch. Inclusion of the competency and quality dimension provided by the physician in future guidelines should be investigated in efforts to improve P-EMS dispatch accuracy. Existing CAD system data is inconsistent and insufficient to provide basic data for scientific research. These factors call for better tools for both dispatch and incident handling for the EMCCs. Future studies are warranted on both validation of Index and in-depth analysis of the core data quality of the applied CAD system. In general, coordination, standardisation, and integration of existing data systems should enhance the quality of trauma care and increase patient safety.

## Supplementary Information

**Additional file 1**. Selected dispatch criteria used as filter in the initial data extraction to identify trauma incidents.

## Data Availability

Data for this study is stored pursuant to the security requirements stated by the Regional Committee for Medical and Health Research Ethics at Oslo University Hospital and at the Medical Faculty, University of Oslo. The unidentified datasets used for analysis for the current study are available through the corresponding author on reasonable request.

## Abbreviations

ACS-COT: American College of Surgeons Committee on Trauma
ALS: Advance life support
AMIS: The emergency medical information system applied by Norwegian EMCCs
CAD: Computer-aided dispatch
DCS: Damage control surgery
ED: Emergency department
EMCC: Emergency medical communication centre
EMS: Emergency medical services
GCS: Glasgow coma scale
GEMS: Ground emergency medical services
HEMS: Helicopter emergency medical services
ICI: Intracranial injury
ISS: Injury severity score
MOI: Mechanisms of injury
NACA: National advisory committee for aeronautics
NISS: New injury severity score
NTR: Norwegian trauma registry
OUH: Oslo University Hospital
P-EMS: Physician-manned emergency medical services
SAR: Search and rescue
STROBE: Strengthening the reporting of observational studies in epidemiology
TBI: Traumatic brain injury
TXA: Tranexamic acid
