# Supplementary material for: Dispatch accuracy of physician-staffed emergency medical services in trauma care in south-east Norway: a retrospective observational study
Source: Scand J Trauma Resusc Emerg Med. 2021 Dec 7;29:169. doi: 10.1186/s13049-021-00982-3 (PMC8650530; doi:10.1186/s13049-021-00982-3)
Supplement: Supplementary file 1 — Additional file 1. Selected dispatch criteria used as filter in the initial data extraction to identify trauma incidents. [file 13049_2021_982_MOESM1_ESM.docx]

**Additional file 1 – Dispatch criteria filter**

| **Criteria group** | **Criteria number** | **Criteria name** |
| --- | --- | --- |
| **Unresponsive adult** | A.01.02 | Cardiac arrest caused by hypoxia or trauma |
| **Major disaster / mass-casualty incident** | A.04.01 | Fire / explosion – incident on land |
|  | A.04.02 | Plane crash |
|  | A.04.03 | Railway / tram / trolleybus accident |
|  | A.04.04 | Road traffic accident |
|  | A.04.05 | Major incident with dangerous goods |
|  | A.04.06 | Avalanche |
|  | A.04.07 | Other natural disaster |
|  | A.04.08 | Gas leakage |
|  | A.04.09 | Shipping accident |
|  | A.04.10 | Accident on offshore installation |
|  | A.04.11 | Major incident with radiation hazard |
|  | A.04.12 | Act of terror / act of war |
|  | A.04.13 | Major incident with dangerous goods / radiation hazard |
|  | A.04.14 | Possible mass-casualty incident |
|  | A.04.15 | Imminent mass-casualty incident |
| **Transport reservations** | A.05.01 | Primary mission. Transport directly to hospital. Unstable vital signs. |
|  | A.05.02 | Primary mission. Transport to higher care level. Unstable vital signs. |
|  | A.05.04 | Police / fire brigade request immediate response |
|  | A.05.06 | Other assignment. Immediate response |
| **Unidentified problem** | A.06.08 | Assume serious / critical problem (more information not readily available) |
|  | A.06.09 | Other serious / critical problem (no adequate criterion elsewhere in Index) |
|  | A.06.10 | Repeated calls, assume serious / critical problem. |
|  | A.06.11 | Difficulty communicating, assume serious / critical problem |
| **Burns / scalding / electrical injury** | A.09.01 | Does not respond to shaking and shouting |
|  | A.09.02 | Breathing problems |
|  | A.09.03 | Semi-conscious / seems drowsy / seems restless |
|  | A.09.04 | Exposed to smoke and difficulty in talking or swallowing |
|  | A.09.05 | Serious burns injury in adult (> 20% of the skin) |
|  | A.09.06 | Serious burns injury in child (> 10% of the skin) |
|  | A.09.07 | Serious burns injury in the face |
|  | A.09.08 | Injury due to high voltage electric current or lightning |
|  | A.09.09 | Other serious / extensive injury in addition to burns |
|  | A.09.10 | Fall over 5 metres |
|  | A.09.11 | Missing persons (ongoing search and rescue operation) |
| **Animal bites / insect stings** | A.14.10 | Major blood loss after bite, still bleeding |
| **Hypothermia / hyperthermia** | A.21.05 | Appears to have a low body temperature with an additional major injury |
| **Chemicals / gasses** | A.22.01 | Leakage of toxic or explosive gases |
|  | A.22.02 | Incident with hazardous chemicals / gases |
|  | A.22.03 | Possible serious injury after incident |
|  | A.22.08 | Major corrosives injury in adult (> 20% of the skin) |
|  | A.22.09 | Major corrosives injury in child (> 10% of the skin) |
|  | A.22.10 | Major corrosives injury in the face / eye |
| **Mental health problems / suicide** | A.28.01 | Does not respond to shaking and shouting |
|  | A.28.02 | Serious suicide attempt with a weapon |
|  | A.28.04 | Serious suicide attempt by knifing / slashing, major blood loss |
|  | A.28.05 | Serious suicide attempt by jump from a great height |
| **Fractures / wounds / minor injuries** | A.33.01 | Breathing problems |
|  | A.33.02 | Suspect a deep cut / knife wound in the throat / chest / abdomen |
|  | A.33.03 | Major blood loss, still bleeding |
|  | A.33.04 | Broken bone ends protruding from the wound |
|  | A.33.05 | Intense pain or deformity in the thigh – possible fracture of the thigh |
|  | A.33.06 | Intense pain in the head / neck / chest / abdomen |
| **Road traffic accident** | A.34.01 | Possible serious injury |
|  | A.34.02 | Possible high-energy injury |
|  | A.34.03 | Person trapped in vehicle |
|  | A.34.04 | Incident with dangerous goods |
|  | A.34.05 | Unconscious – unresponsive / barely responsive |
|  | A.34.06 | Has been unconscious, still feels dizzy |
|  | A.34.07 | Awake, but feels faint |
|  | A.34.08 | Breathing problems |
|  | A.34.09 | Pale and clammy skin |
|  | A.34.10 | Major blood loss, still bleeding |
|  | A.34.11 | Major open wound |
|  | A.34.12 | Severe pain in the head, neck, chest, abdomen, pelvis, thigh |
|  | A.34.13 | Weakness or numbness in legs (suspect neck / spine injury) |
|  | A.34.14 | Danger of severe hypothermia |
| **Accidents** | A.35.01 | Possible serious injury |
|  | A.35.02 | Possible high-energy injury |
|  | A.35.03 | Person trapped |
|  | A.35.04 | Loss of body part |
|  | A.35.05 | Unconscious – unresponsive / barely responsive |
|  | A.35.06 | Has been unconscious, still feels dizzy |
|  | A.35.07 | Awake, but feels faint |
|  | A.35.08 | Breathing problems |
|  | A.35.09 | Pale and clammy skin |
|  | A.35.10 | Major blood loss, still bleeding |
|  | A.35.11 | Major open wound |
|  | A.35.12 | Severe pain in the head, neck, chest, abdomen, pelvis, thigh |
|  | A.35.13 | Weakness or numbness in legs (suspect neck / spine injury) |
|  | A.35.14 | Danger of severe hypothermia |
| **Urinary tracts** | A.36.01 | Major and open injury to kidneys or urinary tract |
| **Violence / abuse** | A.37.01 | Does not respond to shaking and shouting |
|  | A.37.02 | Suspect grave violence or seriously wounded person |
|  | A.37.03 | Awake, but feels faint |
|  | A.37.04 | Breathing problems |
|  | A.37.05 | Stab wound, gunshot wound or other major injury |
|  | A.37.06 | Pale and clammy skin |
|  | A.37.07 | Major blood loss, still bleeding |
|  | A.37.08 | Weakness or numbness in legs (suspect neck / spine injury) |
|  | A.37.09 | Grave danger of serious violence |
| **Eye** | A.39.02 | Major open injury in, or close to, the eye |
|  | A.39.03 | Blow to the eye / head. Awake, but feels faint |
|  | A.39.04 | Major corrosive injury in, or close to, the eye |
